# Dietary determinants of postprandial blood glucose control in adults with type 1 diabetes on a hybrid closed-loop system

**DOI:** 10.1007/s00125-021-05587-0

**Published:** 2021-10-23

**Authors:** Claudia Vetrani, Ilaria Calabrese, Luisa Cavagnuolo, Daniela Pacella, Elsa Napolano, Silvia Di Rienzo, Gabriele Riccardi, Angela A. Rivellese, Giovanni Annuzzi, Lutgarda Bozzetto

**Affiliations:** 1 Department of Clinical Medicine and Surgery, Federico II University, Naples, Italy; 2 Department of Public Health, Federico II University, Naples, Italy

**Keywords:** CGM, Diet composition, Hybrid closed-loop system, Insulin delivery, Postprandial glucose, Type 1 diabetes

## Abstract

**Aims/hypothesis:**

The aim of this work was to assess the relationship between meal nutrients and postprandial blood glucose response (PGR) in individuals with type 1 diabetes on a hybrid closed-loop system (HCLS).

**Methods:**

The dietary composition of 1264 meals (398 breakfasts, 441 lunches and 425 dinners) was assessed by 7-day food records completed by 25 individuals with type 1 diabetes on HCLSs (12 men/13 women, mean ± SD age 40 ± 12 years, mean ± SD HbA_1c_ 51 ± 10 mmol/mol [6.9 ± 0.2%]). For each meal, PGR (continuous glucose monitoring metrics, glucose incremental AUCs) and insulin doses (pre-meal boluses, post-meal microboluses automatically delivered by the pump and adjustment boluses) over 6 h were evaluated.

**Results:**

Breakfast, lunch and dinner significantly differed with respect to energy and nutrient intake and insulin doses. The blood glucose postprandial profile showed an earlier peak after breakfast and a slow increase until 4 h after lunch and dinner (*p* < 0.001). Mean ± SD postprandial time in range (TIR) was better at breakfast (79.3 ± 22.2%) than at lunch (71.3 ± 23.9%) or dinner (70.0 ± 25.9%) (*p* < 0.001). Significant negative predictors of TIR at breakfast were total energy intake, per cent intake of total protein and monounsaturated fatty acids, glycaemic load and absolute amounts of cholesterol, carbohydrates and simple sugars consumed (*p* < 0.05 for all). No significant predictors were detected for TIR at lunch. For TIR at dinner, a significant positive predictor was the per cent intake of plant proteins, while negative predictors were glycaemic load and intake amounts of simple sugars and carbohydrate (*p* < 0.05 for all).

**Conclusions/interpretation:**

This study shows that nutritional factors other than the amount of carbohydrate significantly influence postprandial blood glucose control. These nutritional determinants vary between breakfast, lunch and dinner, with differing effects on postprandial blood glucose profile and insulin requirements, thus remaining a challenge to HCLSs.

**Graphical abstract:**

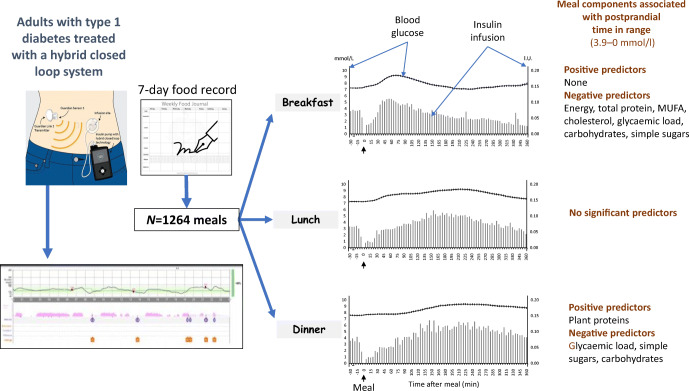

**Supplementary Information:**

The online version of this article (10.1007/s00125-021-05587-0) contains peer-reviewed but unedited supplementary material.

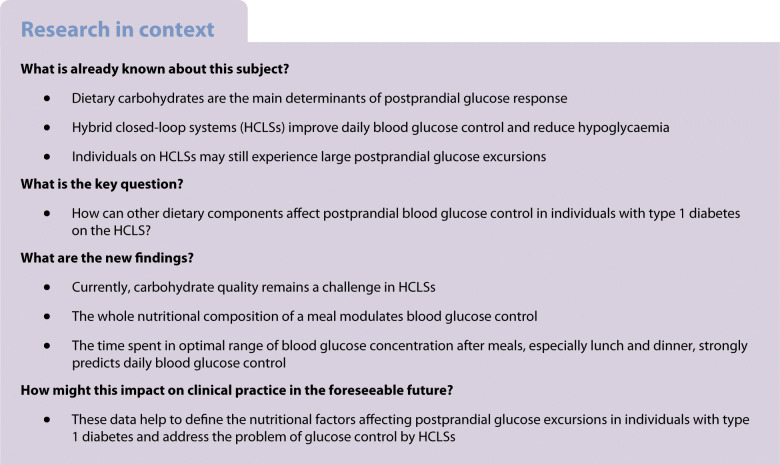



## Introduction

Optimal blood glucose control is challenging in individuals with type 1 diabetes, particularly in the postprandial period [1, 2]. Carbohydrates (CHOs) are the main determinants of postprandial glucose response (PGR) [1]. However, mounting evidence is available on the effects of other dietary components on PGR (i.e. protein and fat). High intake of dietary protein may increase PGR in a dose–response manner [3–5], likely due to the conversion of amino acids into glucose [6]. In addition, the quantity and quality of fats within meals containing the same quantity of CHO may influence the shape and extent of PGR, possibly through the delay of gastric emptying and the reduction of insulin sensitivity [6–8]. A further mechanism for increasing blood glucose levels may relate to glucagon stimulation by protein and fat [9, 10]. This raises concerns on the effectiveness of solely using CHO counting for estimating prandial insulin [11].

On the other hand, CHO counting is essential for the functioning of the most advanced form of insulin delivery available for people with type 1 diabetes, namely, the hybrid closed-loop system (HCLS). While the HCLS offers better overall glucose control and reduced risk of hypoglycaemia [12, 13], users still experience large postprandial glucose excursions due to failures in blunting blood glucose changes after meals [14]. Therefore, postprandial glucose control remains a relevant issue with the present systems of artificial pancreases and needs addressing.

An HCLS automatically delivers basal insulin according to an algorithm based on continuous glucose monitoring (CGM). Additionally, a mealtime bolus of insulin is administered by each individual, after being estimated by the HCLS based on the amount of meal CHO (as entered by the individual) and predefined specific features (insulin sensitivity, postprandial glucose target, insulin on board, insulin/CHO ratio).

There is very little information on the nutritional factors influencing postprandial glucose control in individuals with type 1 diabetes on HCLSs. A recent report showed a better daily glucose control with a lower than a higher CHO intake [15], while no data are available on the effects of the overall meal nutrient composition on daily and postprandial glucose control. This information may be clinically relevant in reducing postprandial glucose excursions, by identifying nutritional determinants and improving HCLSs’ algorithms performance. Therefore, we investigated the association between whole-meal dietary composition and postprandial blood glucose control in individuals with type 1 diabetes on HCLSs under free-living conditions.

## Methods

### Participants

All individuals aged >18 years with type 1 diabetes on an HCLS (MiniMed 670G; Medtronic MiniMed, USA) attending the Diabetes Outpatient Clinic of Federico II University Hospital (Naples, Italy) with at least one 7-day food record available, daily food intake >3350 kJ (800 kcal) and time on HCLS auto-mode during the observation period >97% were eligible for the study. Dietary records included in the analyses were completed from January 2019 to September 2020, accounting for 1264 meals (398 breakfasts, 441 lunches and 425 dinners) (ESM Fig. 1).

Dietary and HCLS records included in the analyses referred to 25 participants (12 men and 13 women, mean ± SD age 40 ± 12 years, mean ± SD diabetes duration 15 ± 11 years, mean ± SD time on HCLS auto-mode duration 157 ± 138 days). They were in satisfactory blood glucose control as shown by HbA_1c_ levels (51 ± 10 mmol/mol [6.9 ± 0.2%]) and daily glucose metrics derived by CGM readings [16] (mean ± SD, time in range [TIR] 3.9–10.0 mmol/l, 75 ± 15%; time above range [TAR] >10.0 mmol/l, 19.4 ± 11%; TAR >13.9 mmol/l, 4.5 ± 6.2%; time below range [TBR] <3.9 mmol/l, 1.2 ± 2.1%; TBR <3.0 mmol/l, 0.3 ± 1.2%).

For the use of her/his data, each participant gave informed consent following the approval of the Ethical Committee of the Federico II University.

### Study design

All participants completed one or more 7-day food diaries after being given descriptive information for identifying foods eaten and guidelines for calculating portion sizes. They recorded all foods and drinks consumed (including dressings) and reported portions by household measures (cups, spoons, etc.) or weight, providing as much detail as possible (i.e. cooking methods, brands names). A skilled dietitian discussed the food records with each participant to check for potential mistakes and missing information. Energy intake, nutrient composition, glycaemic index and glycaemic load were calculated using the MetaDieta software (Meteda, Ascoli-Piceno, Italy). Participants were not requested to report information about physical activity in their food diaries or CGM records. However, during the days of observation, we did not detect any use of temporary blood glucose targets that would indicate a session of physical exercise.

According to HCLS functioning, insulin was administered as a CHO-based pre-meal bolus, post-meal microboluses automatically delivered by the pump and adjustment boluses eventually delivered by the participant according to the HCLS’s suggestion.

Data on glucose concentrations and insulin infusion were obtained from electronic records available on the Medtronic cloud (CareLink; https://carelink.medtronic.eu/). For the analysis of each meal (breakfast, lunch or dinner), glucose and insulin data were collected from 30 min prior to the preprandial insulin bolus and over 6 h thereafter. A time between meals of at least 6 h was observed, although 38 breakfasts (9.5%) with a postprandial observation of only 5.5 h were included in the analysis. Glucose concentrations at 5-min intervals were collected to evaluate incremental AUC (iAUC), calculated using the trapezoidal method and blood glucose control by CGM metrics.

### Statistical analyses

Data are expressed as means ± SDs unless otherwise stated. For each meal, data on blood glucose concentrations and insulin infusion were evaluated as total response (over 6 h after meal), early response (over the first 3 h) and late response (over the last 3 h). Differences in blood glucose or insulin infusion between early and late postprandial phases were evaluated with mixed-effect linear regression considering the participant’s identification number as random effect and the day as a nested random effect. Differences between the type of meal (breakfast, lunch and dinner) in dietary composition, blood glucose iAUC, insulin doses and CGM metrics were analysed using mixed-effect model testing for time interaction. In all analyses, time was considered as a categorical variable. Significant differences were explored with post hoc Bonferroni test for pairwise comparisons. The associations between dietary composition and CGM metrics were assessed by mixed-effect regression considering the participant’s ID as random effect. Mixed-effect models were run separately for each meal to estimate the main effects of meal composition. For all analyses, *p* < 0.05 was considered significant. Statistical analyses were performed according to standard methods using SPSS software version 26 (SPSS/PC; SPSS, Chicago, IL, USA) and R statistical framework version 4.0.3. To fit mixed-effect models, the lmer function in the lme4 R package was used.

## Results

### Meal composition

A total of 1264 meals were analysed: 398 breakfasts, 441 lunches and 425 dinners. The energy content of the three meals was significantly different, breakfast showing the lowest mean energy (900 kJ [215 kcal]) compared with lunch (2426 kJ [579 kcal]) and dinner (2550 kJ [609 kcal]) (Table 1). They significantly differed for the per cent contribution of dietary components to meal’s energy. In particular, breakfast provided the highest percentage of CHO, simple sugar and saturated fatty acids. Lunch provided the highest contribution of plant protein. Dinner showed the highest glycaemic index and glycaemic load. Lunch and dinner were equally richer than breakfast in monounsaturated fatty acids and fibre. The amounts of CHO in meals recorded in the 7-day food diary were very consistent with the CHO values in the CGM/pump reports as entered by the participants for pre-meal bolus calculation (*p* < 0.001).

**Table 1:** Daily energy intake and dietary composition of breakfast, lunch and dinner obtained through 7-day food records

Meal composition	Breakfast (*n* = 398)	Lunch (*n* = 441)	Dinner (*n* = 425)
Energy, kJ (kcal)	900 ± 440 (215 ± 105)	2426 ± 820* (579 ± 196)	2550 ± 1021*^†^ (609 ± 244)
Protein, %TEI	17.7 ± 7.4	16.4 ± 6.6*	20.8 ± 9.3*
Animal origin	14.5 ± 8.1	6.9 ± 7.7*	14.2 ± 9.7^†^
Plant origin	2.4 ± 4.5	8.7 ± 3.6*	5.9 ± 3.2*^†^
CHO, %TEI	55.6 ± 12.3	51.4 ± 13.1*	45.4 ± 15.6*^†^
Simple sugar, %TEI	29.0 ± 14.0	10.2 ± 8.1*	11.0 ± 8.4*
Fat, %TEI	26.9 ± 9.3	32.3 ± 10.8*	33.9 ± 13.4*
SFA	12.2 ± 5.4	6.9 ± 4.8*	8.4 ± 5.2*^†^
MUFA	7.9 ± 4.0	16.3 ± 6.5*	15.9 ± 7.4*
PUFA	3.2 ± 2.6	4.5 ± 2.9*	4.2 ± 2.8*
Cholesterol, mg	21.4 ± 20.9	58.9 ± 86.0*	89.1 ± 95.4*
Fibre, g	1.6 ± 1.8	8.1 ± 5.0*	7.8 ± 4.6*
Glycaemic index, %	51.4 ± 11.7	52.8 ± 11.4	63.1 ± 11.6*^†^
Glycaemic load, units	16.6 ± 8.8	39.4 ± 14.4*	46.4 ± 30.1*^†^

Data are presented as means ± SD

*p < 0.05 vs breakfast; ^†^*p* < 0.05 vs lunch (mixed-effect model with post hoc Bonferroni test for multiple comparisons)

MUFA, monounsaturated fatty acids; PUFA, polyunsaturated fatty acids; SFA, saturated fatty acids; TEI, total energy intake

### Postprandial blood glucose

**Fig. 1:**
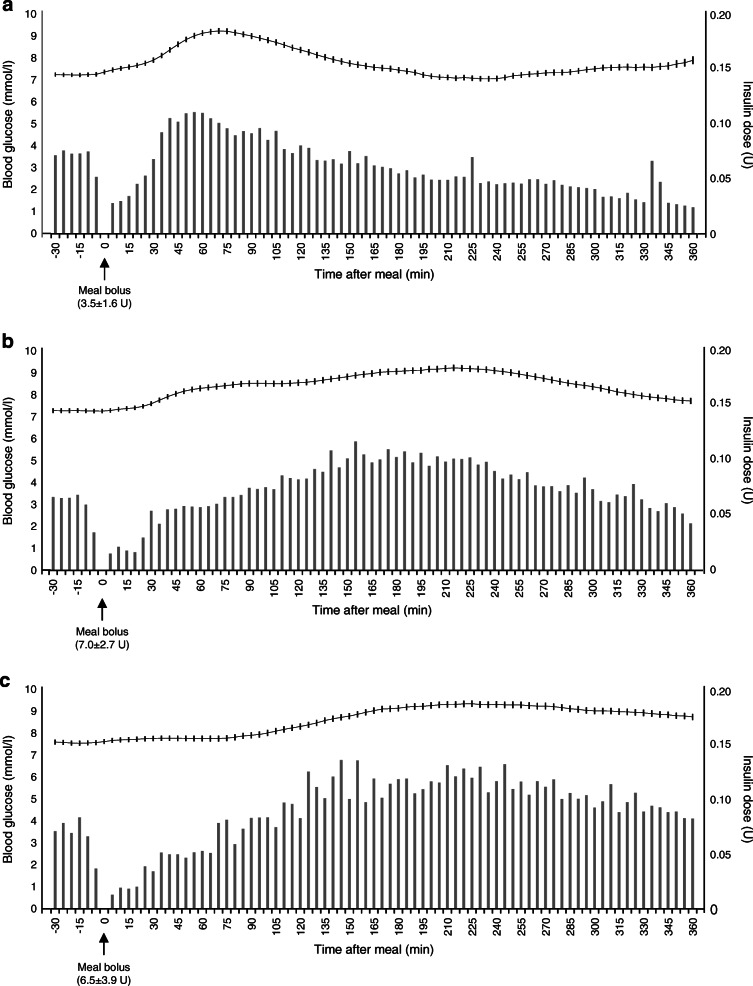
Blood glucose concentrations (continuous line, mean ± SE) and insulin doses delivered by the pump automatically as post-meal microboluses and as adjustment boluses delivered by the participant according to HCLS suggestion (vertical bars) at breakfast (*n* = 398) (**a**), lunch (*n* = 441) (**b**) and dinner (*n* = 425) (**c**)

Blood glucose postprandial profile differed by type of meal (Fig. 1). Mixed-effect regression showed that glucose iAUCs differed between the early (0–3 h) and late (3–6 h) postprandial phase at breakfast (*p* < 0.001), lunch (*p* < 0.001) and dinner (*p* < 0.001). Descriptively, after breakfast, there was an early rise in blood glucose peaking after just 1 h, with an iAUC_0–3 h_ higher than iAUC_3–6 h_ (165 ± 408 vs −9.6 ± 468 mmol/l×180 min). On the contrary, at dinner, mean glucose concentrations slowly increased until 4 h after the meal, with higher iAUC_3–6 h_ than iAUC_0–3 h_ (237 ± 534 vs 101 ± 382 mmol/l×180 min).

**Fig. 2:**
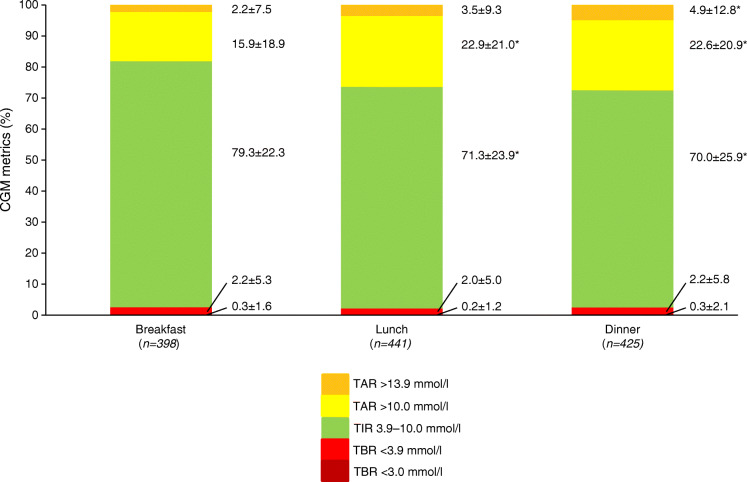
CGM metrics at breakfast, lunch and dinner. Means ± SD; *p < 0.05 vs breakfast (mixed-effect model with post hoc Bonferroni test for multiple comparisons)

Postprandial blood glucose control evaluated by CGM metrics was generally adequate after each type of meal, with a TIR >70% and a TBR <2.5% (Fig. 2). Postprandial glucose control was better at breakfast (TIR 79.3 ± 22.2%) than lunch (71.3 ± 23.9%) or dinner (70.0 ± 25.9%) (*p* < 0.001). Severe hyperglycaemia (TAR >13.9 mmol/l) was more frequent at dinner than lunch and breakfast.

Daily TIR was significantly associated with breakfast TIR (estimated coefficient of the mixed effect of the regression model [Est.] = 0.258, *p* < 0.001), lunch TIR (Est. = 0.290, *p* < 0.001) and dinner TIR (Est. = 0.194, *p* < 0.001).

### Insulin doses

Pre-meal insulin boluses at breakfast were lower than at lunch and dinner (*p* < 0.001) (Fig. 1). The pattern of insulin infusion after a meal differed significantly by the type of meal (*p* < 0.001, time × meal interaction). Insulin doses after breakfast were higher during the first 3 h (2.7 ± 1.8 U) than during the last 3 h (1.7 ± 1.9 U, *p* = 0.016); on the contrary, the doses were lower during the first 3 h than during the last 3 h after lunch (2.6 ± 2.1 vs 2.9 ± 2.1 U, *p* = 0.005) and dinner (2.8 ± 2.2 vs 3.8 ± 2.4 U, *p* = 0.009).

### Relationships between meal composition and postprandial glucose control

The associations between postprandial glucose TIR and meal composition are reported in Table 2. At breakfast, TIR was significantly associated inversely with the intake of energy (*p* = 0.01), per cent intake of total protein (*p* = 0.02) and monounsaturated fatty acids (*p* = 0.02), glycaemic load (*p* = 0.004) and the amounts of cholesterol (*p* = 0.01), CHO (*p* = 0.005) and simple sugars (*p* < 0.001) in the meal. At lunch, no significant associations were detected between dietary composition and TIR. At dinner, TIR was significantly associated directly with per cent intake of protein of plant origin (*p* = 0.04) and inversely with glycaemic load (*p* = 0.03), per cent intake of simple sugars (*p* = 0.002) and amount of CHO in the meal (*p* = 0.04).

**Table 2:** Associations between meal nutrient composition and blood glucose control (TIR 3.9–10.0 mmol/l) at breakfast, lunch and dinner

Meal composition	Breakfast TIR		Lunch TIR		Dinner TIR	
	Est.	*p* value	Est.	*p* value	Est.	*p* value
Energy, kcal	−0.04	0.01*	−0.001	0.90	−0.009	0.08
Protein, %TEI	−0.99	0.02*	0.13	0.16	0.02	0.84
Animal origin	−0.17	0.40	0.12	0.43	0.21	0.10
Plant origin	0.23	0.41	0.27	0.39	0.80	0.04*
CHO, %TEI	0.02	0.85	−0.13	0.15	−0.13	0.09
CHO, g	−0.29	0.005*	−0.03	0.54	−0.07	0.04*
Simple sugar, %TEI	0.02	0.79	−0.30	0.06	−0.38	0.02*
Simple sugar, g	−0.717	<0.001*	−0.04	0.73	−0.300	0.002*
Fat, %TEI	−0.04	0.77	0.06	0.55	0.08	0.41
SFA	−0.36	0.15	−0.07	0.78	0.0002	0.99
MUFA	−0.75	0.02*	0.03	0.88	0.28	0.09
PUFA	−0.43	0.33	0.60	0.14	0.54	0.22
Cholesterol, mg	−0.17	0.01*	0.001	0.93	−0.006	0.65
Fibre, g	−0.07	0.93	0.16	0.50	0.16	0.60
Glycaemic index, %	−0.06	0.65	0.009	0.93	−0.07	0.56
Glycaemic load, units	−0.52	0.004*	−0.08	0.31	−0.09	0.03*

*p < 0.05

MUFA, monounsaturated fatty acids; PUFA, polyunsaturated fatty acids; SFA, saturated fatty acids; TEI, total energy intake

The associations between meal composition and early or late post-meal insulin dose (microboluses automatically delivered by the pump plus adjustment boluses delivered by the participant according to HCLS suggestion) are reported in Table 3. At breakfast, early (0–3 h) insulin delivery was inversely associated with the energy content of the meal (Est. = −0.003, *p* = 0.03), intake of protein (Est. = −0.07 for per cent of total energy intake, *p* = 0.04) and CHO (Est. = −0.02 for absolute intake [g], *p* = 0.04) and the glycaemic load (Est. = −0.03, *p* = 0.02). Late (3–6 h) insulin delivery was inversely associated with glycaemic index (Est. = −0.04, *p* = 0.002) and glycaemic load (Est. = −0.04, *p* = 0.03). At lunch, early (0–3 h) insulin delivery was inversely associated with the intake of polyunsaturated fatty acids (Est. = −0.07, *p* = 0.04). Late (3–6 h) insulin delivery at lunch was directly associated with meal energy (Est. = 0.001, *p* = 0.04) and content of simple sugars (g) (Est. = 0.02, *p* = 0.02), while there was an inverse association with glycaemic index (Est. = −0.02, *p* = 0.047). At dinner, no significant predictors of early insulin delivery were identified. Conversely, late (3–6 h) insulin delivery was inversely associated with the meal energy (Est. = 0.002, *p* < 0.001) and the content of CHO (g) (Est. = 0.01, *p* = 0.001) and simple sugars (g) (Est. = 0.02, *p* = 0.006) and glycaemic load (Est. = 0.01, *p* = 0.002).

**Table 3:** Associations between meal nutrient composition and post-meal insulin doses (microboluses automatically delivered by the pump plus adjustment boluses delivered by the participants according to HCLS suggestion) at breakfast, lunch and dinner in the early (0–3 h) and late (3–6 h) postprandial phases

Meal composition	Insulin after breakfast				Insulin after lunch				Insulin after dinner			
	0–3 h		3–6 h		0–3 h		3–6 h		0–3 h		3–6 h	
	Est.^a^	*p* value	Est.^a^	*p* value	Est.^a^	*p* value	Est.^a^	*p* value	Est.^a^	*p* value	Est.^a^	*p* value
Energy (kcal)	−0.003	0.03*	−0.002	0.16	0.00001	0.94	0.001	0.04*	−0.0004	0.41	0.002	<0.001*
Protein, %TEI	−0.07	0.04*	0.02	0.66	−0.005	0.52	0.007	0.30	−0.0004	0.95	0.01	0.18
Animal origin	0.01	0.34	0.06	<0.001*	−0.009	0.50	0.01	0.25	0.002	0.87	−0.003	0.78
Plant origin	−0.01	0.57	−0.04	0.09	0.007	0.81	−0.02	0.37	−0.0005	0.99	−0.04	0.21
CHO, %TEI	−0.001	0.89	−0.01	0.26	0.006	0.41	−0.003	0.66	−0.004	0.55	0.007	0.34
CHO, g	−0.02	0.04*	−0.02	0.09	0.002	0.59	0.004	0.27	−0.003	0.33	0.01	0.001*
Simple sugar, %TEI	0.01	0.12	−0.001	0.89	0.02	0.13	0.02	0.15	0.007	0.60	0.008	0.59
Simple sugar, g	−0.02	0.28	−0.02	0.24	−0.01	0.33	0.02	0.02*	0.002	0.84	0.02	0.006*
Fat, %TEI	−0.001	0.94	−0.0006	0.95	−0.006	0.52	0.003	0.77	0.003	0.72	−0.005	0.57
SFA	0.03	0.11	0.03	0.15	−0.02	0.28	0.02	0.35	0.01	0.53	−0.01	0.56
MUFA	0.02	0.47	0.003	0.89	−0.02	0.28	−0.02	0.15	0.009	0.52	−0.02	0.22
PUFA	−0.001	0.97	−0.05	0.14	−0.07	0.04*	0.03	0.30	0.01	0.69	0.008	0.84
Cholesterol, mg	0.00001	0.99	−0.003	0.57	−0.0009	0.38	0.001	0.24	0.0004	0.68	−0.001	0.29
Fibre, g	−0.100	0.09	−0.08	0.25	−0.006	0.77	−0.006	0.77	−0.03	0.17	0.02	0.39
Glycaemic index, %	−0.01	0.37	−0.04	0.002*	−0.002	0.80	−0.02	0.047*	−0.02	0.09	0.01	0.29
Glycaemic load, units	−0.03	0.02*	−0.04	0.03*	0.007	0.32	0.0007	0.92	−0.004	0.22	0.01	0.002*

MUFA, monounsaturated fatty acids; PUFA, polyunsaturated fatty acids; SFA, saturated fatty acids; TEI, total energy intake

^a^Regression coefficients are adjusted for all other meal components (models were fitted for each combination of meal and phase)

*p < 0.05

## Discussion

This is the first study to evaluate the relationship between the full nutrient composition of meals and postprandial glycaemic response in individuals with type 1 diabetes on HCLSs. It shows, in a large series of observations in free-living conditions, that nutritional factors other than the amount of CHO significantly influence postprandial blood glucose control. These nutritional effects vary between breakfast, lunch and dinner, differently affecting postprandial blood glucose shape.

The study participants were on optimal blood glucose control (TIR 75 ± 15%) according to levels recommended [16] or reported in other populations [17, 18]. They also showed an optimal adherence to CHO counting as suggested by the good correspondence between the CHO intake reported in the food diary and the amount of CHO stated in the pump reports. This resulted in a good management of the CHO content of the meal, making it easier to bring to light the role of nutrients other than CHOs in postprandial glucose profiles.

At breakfast, there were significant relationships between nutritional factors and postprandial blood glucose control; a higher glycaemic load was a predictor of decreased postprandial TIR, driven by a main contribution of simple sugars. This may indicate that the HCLS algorithm was unable to compensate for the CHO quality, as suggested by the significant predictive value of the amount of simple sugars consumed. This is in line with the inverse association between glycaemic load and insulin doses administered after the meal, which were lower than actually needed, likely because the algorithm took into account the insulin on board originated by the preprandial insulin bolus. The insulin doses given by the system in response to the intake of simple sugars did not control glucose fluctuations effectively, as shown by the lack of increase in insulin delivery in relation to the intake of simple sugars, shaping an inadequate reaction to the steep and early increase in blood glucose levels. Therefore, adapting pre-meal insulin dose and timing to the glycaemic load of the meal rather than to the CHO content [19] could help the algorithm to providing correction boluses able to properly react to the intake of foods with a high glycaemic index. On the other hand, from a nutritional point of view, this evidence strongly indicates that, taking into account the limitations of the available algorithms for insulin delivery, it could be wise to modify the composition of breakfast by reducing simple sugars and increasing dietary fibre.

Dinner showed the worst postprandial control compared with the other meals, with a glucose response increasing up to 6 h after the meal. This was generally the meal highest in protein and fat; therefore, our observations are in line with previous reports demonstrating that meals rich in fat and/or protein delay the rise of postprandial glucose concentrations [3, 8, 20]. As dinner was also the meal with the highest glycaemic index and glycaemic load, we can speculate that the late increases in postprandial glucose are more evident in the context of fat/protein meals in which the CHOs have a high glycaemic index, since all these features of the meal contribute to an increased blood glucose response. In this respect, it is of note that the only significant positive predictor of dinner TIR was the intake of plant proteins, likely due to an increased intake of foods with a low glycaemic index (e.g. legumes or whole grains).

We did not observe any relationship between post-lunch TIR and meal composition. The greater variety of foods used for this meal may have amplified the possible interactions between different dietary components, thus helping to conceal the influence of single nutrients.

It is of note that total energy intake predicted the impairment of the postprandial blood glucose control. Therefore, in addition to meal nutrient composition, the role of the meal size should be considered when predicting postprandial blood glucose.

In our study, postprandial TIR significantly influenced overall glucose control expressed by daily TIR, thus also confirming the clinical relevance of postprandial glucose control in the context of HCLSs. This may especially concern lunch and dinner, considering the different shapes of blood glucose response curves after the different types of meal (i.e. glucose elevations lasted up to 6 h after lunch and dinner while generally expiring 3 h after breakfast).

The clinical implications of the results of this study are that the contribution made by the absolute amount of CHO to postprandial glucose control is well-managed by well-trained users of CHO counting among individuals on HCLS. However, dealing with the early impact of simple sugars or the delayed effects of fat and protein is still challenging due to the peculiar shapes of postprandial responses that are not manageable by the reactive approach typical of the algorithms of first-generation HCLSs. It is not known whether the new more advanced systems, with algorithms including automatic correction boluses, may react more efficiently to the overall meal composition. For the time being, beyond accurate CHO/glycaemic load counting, nutritional education should focus on limiting intake of foodstuffs rich in simple sugars and fat and increasing intake of foods with a low glycaemic index and high fibre content.

Our study has several strengths, including the large number of meals evaluated. Moreover, nutritional data were obtained by using the weighted 7-day food records that represent the gold standard for assessing dietary composition at individual level [21]. In addition, food records were collected within a wide time range and in free-living condition, inducing a huge variety in food choices related to seasonal changes and working days/holidays.

A limitation in our study is that the information regards Italian eating habits, characterised by a small-size sweet breakfast and two main meal courses. However, the separate analysis of the meals allows translation of the information to meals with similar composition independently of when they are consumed (i.e. Italian breakfast resembles the composition of multiple snacking in westernised dietary patterns). Another limitation is the possible underreporting that is common to all types of food recording. However, all food records were discussed with a skilled dietitian to check for potential errors.

In conclusion, the present study provides evidence that not only the amount of CHOs but also the whole nutritional composition of the meal modulates blood glucose control in individuals with type 1 diabetes on an HCLS. Therefore, a comprehensive nutritional education is a key factor to optimise blood glucose control in individuals with type 1 diabetes even in the era of advanced technologies. In addition, our findings highlight the need for better performing algorithms based on nutrient sensing and individual nutrient responsiveness that could allow prediction rather than reaction-driven insulin delivery.

## Supplementary information

ESM (PDF 175 kb)

## Data Availability

The datasets generated during and/or analysed during the current study are available from the corresponding author on reasonable request.

## Abbreviations

CGM: Continuous glucose monitoring
CHO: Carbohydrate
Est.: Estimated coefficient of the mixed effect of the regression model
HCLS: Hybrid closed-loop systems
iAUC: Incremental AUC
PGR: Postprandial glucose response
TAR: Time above range
TBR: Time below range
TIR: Time in range
