# Supplementary material for: Dietary determinants of postprandial blood glucose control in adults with type 1 diabetes on a hybrid closed-loop system
Source: Diabetologia. 2021 Oct 23;65(1):79–87. doi: 10.1007/s00125-021-05587-0 (PMC8660714; doi:10.1007/s00125-021-05587-0)
Supplement: Supplementary file 1 — (PDF 175 kb) [file 125_2021_5587_MOESM1_ESM.pdf]

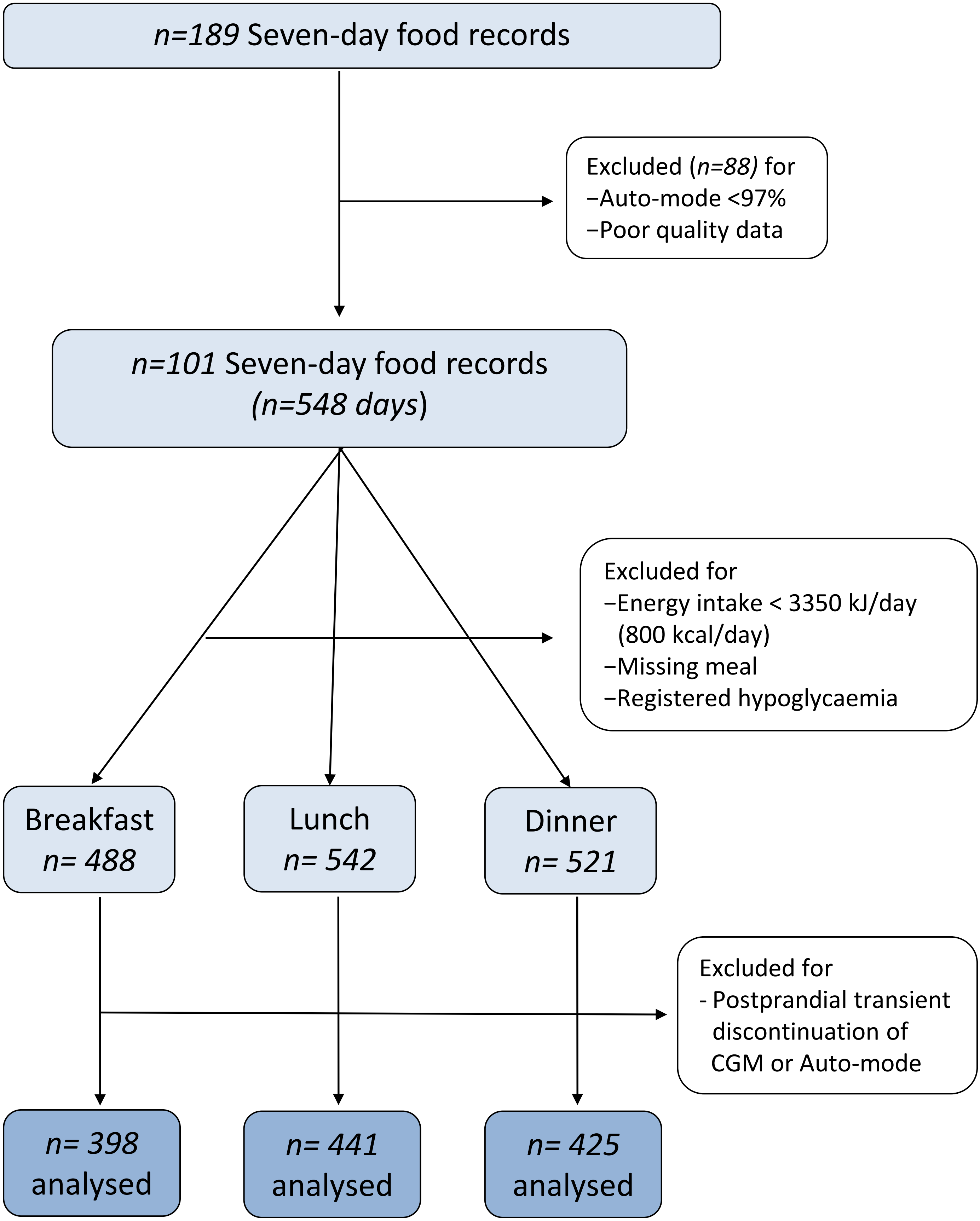

ESM Figure 1. Flow chart of 7-day food records retrieval and selection strategy for the identification of meals to be included in the analyses.
